# The role of protease-activated receptor-2 on pulmonary neutrophils in the innate immune response to cockroach allergen

**DOI:** 10.1186/1476-9255-9-32

**Published:** 2012-09-06

**Authors:** Riad Lutfi, Ian P Lewkowich, Ping Zhou, John R Ledford, Kristen Page

**Affiliations:** 1 Department of Pediatrics, Division of Critical Care Medicine, Cincinnati, OH, USA; 2 Immunobiology, Cincinnati Children’s Hospital Medical Center, Cincinnati, OH, USA

## Abstract

**Background:**

Serine proteases in German cockroach (GC) have been shown to mediate allergic airway inflammation through the activation of protease activated receptor (PAR)-2. Neutrophils play an important role in regulating the innate immune response, and are recruited into the airways following GC frass exposure. As such, we investigated the role of PAR-2 in airway neutrophil recruitment, activation and cytokine production following allergen exposure.

**Methods:**

Wild type and PAR-2-deficient mice were administered a single intratracheal instillation of PBS or GC frass and neutrophil recruitment, expression of PAR-2, CD80, CD86, and MHC class II were assessed by flow cytometry and levels of tumor necrosis factor (TNF)α was assessed by ELISA. Uptake of AlexaFluor 405-labeled GC frass by neutrophils was performed by flow cytometry.

**Results:**

Neutrophil recruitment in the lung and airways following GC frass exposure was significantly decreased in PAR-2-deficient mice compared to wild type mice. GC frass exposure increased the level of PAR-2 on pulmonary neutrophils and increased numbers of PAR-2-positive neutrophils were found in the lungs; however PAR-2 did not play a role in meditating allergen uptake. Comparing wild type and PAR-2-deficient mice, we found that a single exposure to GC frass increased levels of CD80 and CD86 on pulmonary neutrophils, an effect which was independent of PAR-2 expression. Neutrophils isolated from the whole lungs of naïve PAR-2-deficient mice treated *ex vivo* with GC frass produced significantly less TNFα than in similarly treated wild type neutrophils. Lastly, neutrophils were isolated from the bronchoalveolar lavage fluid of wild type and PAR-2-deficient mice following a single intratracheal exposure to GC frass. Airway neutrophils from PAR-2-deficient mice released substantially decreased levels of TNFα, suggesting a role for PAR-2 in neutrophil-derived cytokine production.

**Conclusions:**

Together these data suggest PAR-2 expression can be upregulated on lung neutrophils following allergen exposure and the consequence is altered release of TNFα which could drive the early innate immune response.

## Background

Asthma is regarded as a chronic inflammatory disease of the airways, characterized by airway hyperresponsiveness, airway inflammation and excessive mucus production. Many cell types are involved in the pathophysiology of allergic airway inflammation including eosinophils, mast cells and lymphocytes. Neutrophils are also associated with asthma, and it has been shown that patients with acute [1] or persistent asthma [2] have increased neutrophil levels compared with controls. In some cases, neutrophil levels instead of eosinophil levels have been shown to more closely correlate with airway obstruction and the severity of asthma [3]. In our murine model of German cockroach (GC) feces (frass)-mediated allergic airway inflammation, we find significantly increased levels of neutrophils in the bronchoalveolar lavage (BAL) fluid [4,5]. Others have shown an early and transient increase of neutrophils into the airways following allergen exposure in humans and OVA challenge in mice [6]. Considering the fact that neutrophils play an immediate role in host defense, it seems reasonable that they may be poised to control the events leading up to the generation of adaptive immunity.

We have recently shown an early and substantial increase in airway neutrophilia following a single exposure to the allergen, GC frass [7]. GC frass contains serine proteases that activate protease-activated receptor (PAR)-2 on a variety of cells including human bronchial epithelial cells [8] mouse tracheal epithelial cells [9] and alveolar macrophages [10]. PAR-2 is a G-coupled protein receptor that is activated upon cleavage by a number of extracellular proteases. This allows the protease to signal directly to cells via the cleavage and activation of the receptors on the cell surface. PAR-2 is expressed by many cells in the lung and has been implicated in mediating allergic airway inflammation [4]. We have recently shown that airway neutrophilia is induced following a single exposure to GC frass, and is partially dependent on the activation of PAR-2 [10]. PAR-2 has been shown to be expressed on human neutrophils [11], however the importance of PAR-2 in the activation of neutrophils in the airways following allergen exposure is currently unknown. Since neutrophils are poised to regulate the innate and adaptive immune response, we queried the expression of PAR-2 on airway neutrophils following allergen exposure, and the importance of PAR-2 activation in the context of the innate immune response to allergen exposure. Our findings suggest that there may be a defect in neutrophil recruitment into the airway spaces in PAR-2-deficient mice. In addition, PAR-2 does not play a role in allergen uptake or co-stimulatory molecule expression on the neutrophil, but does play an important role in the regulation of neutrophil-derived TNFα production.

## Methods

### German cockroach frass

The fecal remnants (frass) from one cage of German cockroaches were transferred to a sterile container and stored at 4°C. GC frass was resuspended in endotoxin-free double-distilled water (2 h at 4°C while rocking). Extracts were centrifuged to remove debris (10,000 g for 10 min at 4°C), supernatants harvested, and total protein was measured using the Bio-Rad Protein Assay Dye (Bio-Rad, Hercules, CA). GC frass was frozen in aliquots for use throughout the entire study. AlexaFluor-405 (Invitrogen, Carlsbad, CA) labeled GC frass (AF405-GC frass) was made according to manufacturers’ specifications.

### Animals and GC frass exposure

BALB/c and PAR-2-deficient mice were obtained from Jackson Laboratory (Bar Harbor, ME). PAR-2-C57Bl/6 mice were backcrossed for 10 generations onto the BALB/c background [4]. A single exposure to PBS or GC frass was followed by a lethal dose of sodium pentobarbital 20 h later. AlexaFluor-405 (Invitrogen, Carlsbad CA) labeled GC frass (AF405-GC frass) was made according to manufacturers’ specifications and was used to expose mice to a trackable antigen. These studies were approved by the Cincinnati Children’s Hospital Medical Center Institutional Animal Care and Use Committee.

### Differential cell count and BALF ELISAs

Following PBS or allergen exposure, lungs were lavaged with Hanks balanced salt solution without calcium or magnesium. The lavage fluid was centrifuged (300 g for 10 min at 4°C), and the supernatant was removed. The cell pellet was resuspended in 1 ml of 10% fetal bovine serum in PBS. Total cell numbers were counted on a hemocytometer and 200 μl of the resusupended bronchoalveolar lavage (BAL) cells were centrifuged onto a microscope slide using the Cytospin II centrifuge (Shandon Thermo, Waltham, MA) for 10 min at 64 g at room temp. Once dried, cells were stained with Diff-Quick (Thermo Electron, Pittsburgh, PA) solution for differential cell staining. The BAL fluid (BALF) was analyzed for KC and macrophage inflammatory protein (MIP)-2 by ELISA (R&D Systems, Minneapolis, MN).

### Flow cytometry

Following exposure, whole mouse lungs were diced and placed in RPMI 1640 containing Liberase CI (0.5 mg/ml; Roche Diagnostics, Indianapolis, IN) and DNase I (0.5 mg/ml; Sigma, St. Louis MO) at 37°C for 45 min. The tissue was forced through a 70-micron cell strainer, and red blood cells were lysed with ACK lysis buffer (Invitrogen, Carlsbad, CA). Cells were washed with RPMI containing 10% FBS, counted and 5× 10^5^ cells were used for staining. Staining reactions were performed at 4°C following incubation with Fc block (mAb 2.4 G2) for 30 min. Neutrophils (CD11c+, CD11b+, Gr1+) were quantified using anti-CD11c-APC (HL3), anti-CD11b-PE-Cy7 (M1/70), and anti Gr-1-APC-Cy7(RB6-8C5). Co-stimulatory molecule expression was examined using PE-conjugated mAbs to CD86 (GL1), CD80 (16-10A1), and MHC class II (I-A/I-E). PAR-2 expression was examined using a PE-conjugated mAb to PAR-2. Dead cells were excluded using 7-AAD. All antibodies were purchased from eBioscience (San Diego, CA), with the exception of the PAR-2 mAb (Santa Cruz, Santa Cruz, CA). Data were acquired with an LSRII flow cytometer (BD Biosciences, San Jose, CA). Spectral overlap was compensated using the FACSDiVa software (BD Biosciences) and analyzed using FlowJo software (Treestar Inc, Ashland, OR).

### Assessment of neutrophil-derived cytokine expression

This was performed two ways. First, whole lungs from untreated wild-type or PAR-2-deficient mice were isolated and single lung cell suspensions were made by incubating the minced lungs in Liberase/DNase I as described for flow cytometry. Resuspended cells were layered onto a three-step Percoll gradient (52, 64, and 72%) and centrifuged (300 g x for 30 min at room temp). Neutrophils contained in the bottom layer (64–72%) were collected, counted and plated as previously shown [7]. Wild type and PAR-2-/- neutrophils (1 × 10^6^ cells per well) were treated *ex vivo* with PBS or GC frass (1 μg/ml) for 18 h. Cell supernatants were isolated, clarified and analyzed by ELISA. Second, groups of wild type and PAR-2-/- mice were treated with a single exposure of GC frass (40 μg/40 μl). 18 h later, BAL fluid was harvested and neutrophils were isolated using the Percoll gradient as indicated above. Cells (5 × 10^5^) were incubated for an additional 18 h to allow for cytokine release, but there was no further treatment of these cells. There were no mice treated with PBS in this case because there would not be enough neutrophils in the BAL fluid following this treatment. In all cases, supernatants were analyzed for TNFα production by ELISA (R&D Systems, Minneapolis, MN).

### Statistical analysis

When applicable, statistical significance was assessed by Students t-test or one-way analysis of variance (ANOVA). Differences identified by ANOVA were pinpointed by Student-Newman-Keuls’ multiple range test using SigmaStat software.

## Results

### GC frass induced neutrophil recruitment into the lung

**Figure 1:**
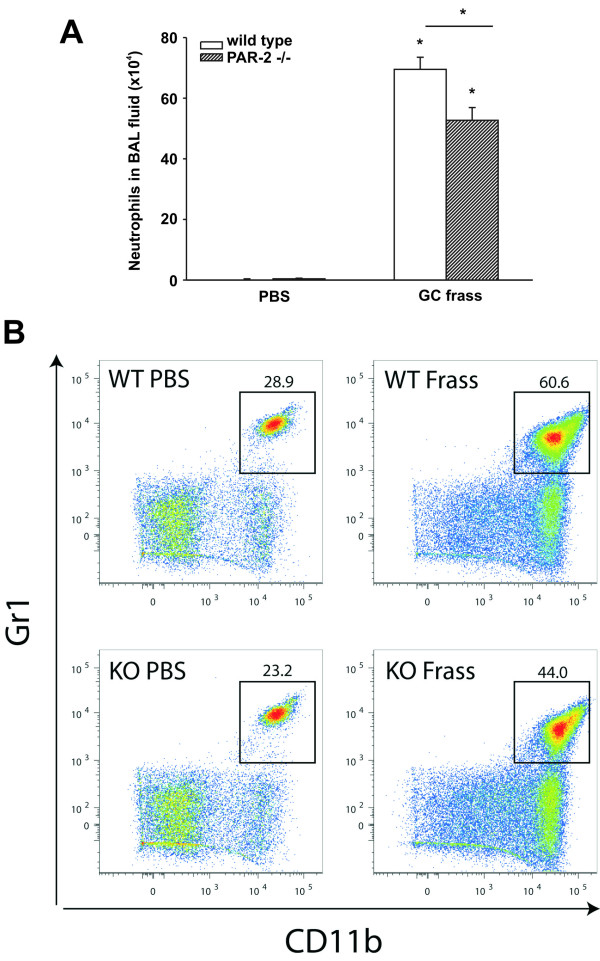
**Neutrophil recruitment into the lung and BAL fluid following allergen challenge.** Naïve BALB/c mice were administered a single intratracheal instillation of PBS or GC frass (40 μg/40 μl) and 20 h later, mice were either lavaged to isolate BAL cells or whole lungs were digested and analyzed for neutrophil content by flow cytometry. **A**. BAL fluid was harvested and differential cell counts performed. Data represent 8 mice per group and are expressed as total cell number mean ± SEM. Statistical significance was determined by ANOVA (*p < 0.001). **B**. Cells were dissociated from whole lungs and stained for flow cytometry analysis. CD11c + cells were subsequently analyzed for CD11b and Gr1 expression. Data are from a single mouse per group but are representative of 6 mice in each group.

To confirm that a single exposure to GC frass induced airway neutrophilia, mice were administered an intratracheal instillation of GC frass and 18 h later, BAL fluid was harvested. We found that a single exposure to GC frass induced significant neutrophilia in the lungs of wild-type mice, there was considerably less neutrophilia in the BAL fluid from PAR-2-deficient mice (Figure 1A). To determine the level of neutrophils in the whole lung, we administered GC frass and harvested the lungs 18 h later for flow cytometry. Compared to PBS-treated mice, GC frass exposure increased the overall number of neutrophils in the lungs of both wild type and PAR-2-deficient mice. The level of recruitment of neutrophils into the lungs of PAR-2-deficient mice was lower than in wild type mice (Figure 1B). We have previously shown that PAR-2-deficient mice had decreased release of KC, a potent neutrophil chemoattractant following GC frass exposure [10]. Since MIP-2 is also chemotactic for neutrophils, we asked whether GC frass increased MIP-2 expression and if this was regulated by PAR-2 expression. Interestingly, while GC frass exposure increased MIP-2 expression (119.5 ± 8.3 ng/ml following GC frass treatment compared to PBS-treated 62.1 ± 2.6 ng/ml, p < 0.001 n = 9–12 mice per group), there was no difference in MIP-2 levels in PAR-2-deficient mice following GC frass treatment (120.7 ± 9.6 ng/ml, p = 0.9). Levels of MIP-2 in PAR-2-deficient mice with PBS exposure (59.6 ± 1.4 ng/ml) were also comparable to wild type. These data suggest that neutrophil recruitment into the airways of mice is partially dependent on the presence of PAR-2, but may also be regulated by the reduced levels of KC production in the PAR-2-deficient mice.

### Regulation of PAR-2 expression on pulmonary neutrophils and uptake of allergen

**Figure 2:**
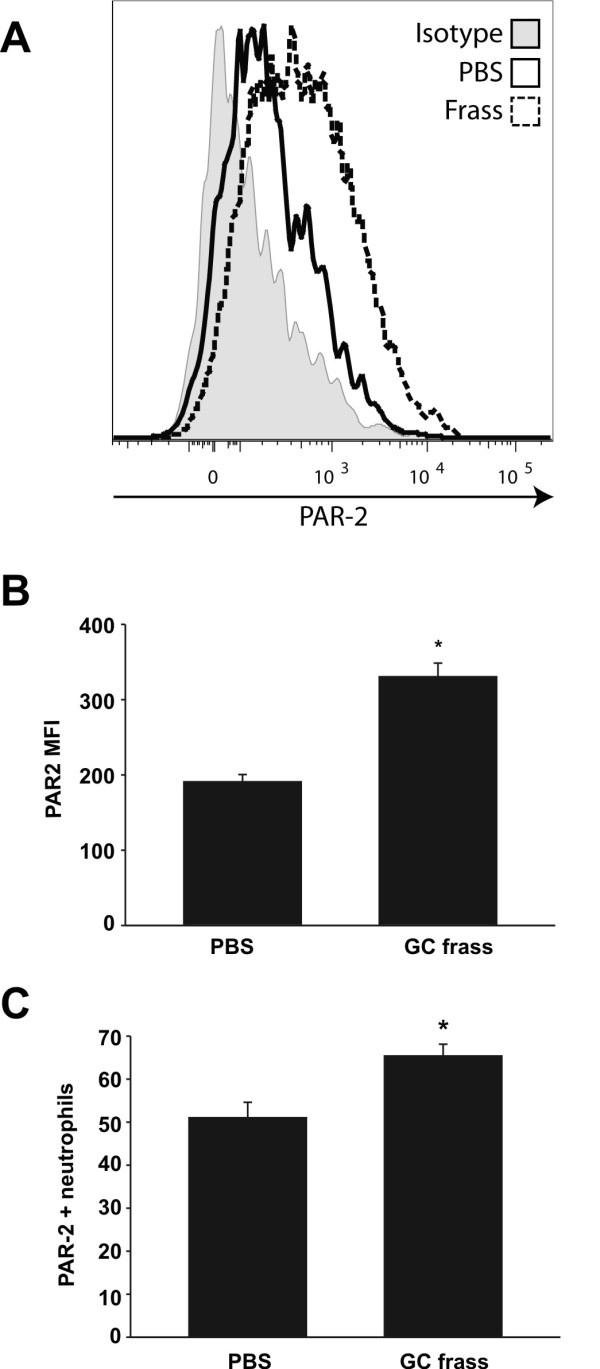
**GC frass increased PAR-2 expression on pulmonary neutrophils.** Lungs from PBS or GC frass-treated mice were harvested 20 h post exposure and stained for flow cytometric analysis of PAR-2 expression. **A**. Representative histogram showing PAR-2 expression on gated pulmonary neutrophils from PBS-treated (solid line) or GC frass-treated (broken line) mice. Solid grey histogram depicts staining with isotype control antibody. **B**. Average PAR-2 MFI. **C**. Percentage of PAR-2-positive neutrophils. In both cases (B + C), data are expressed as mean ± SEM (n = 8 mice per group) and statistical significance was determined by Student’s t-test (*p < 0.001).

**Figure 3:**
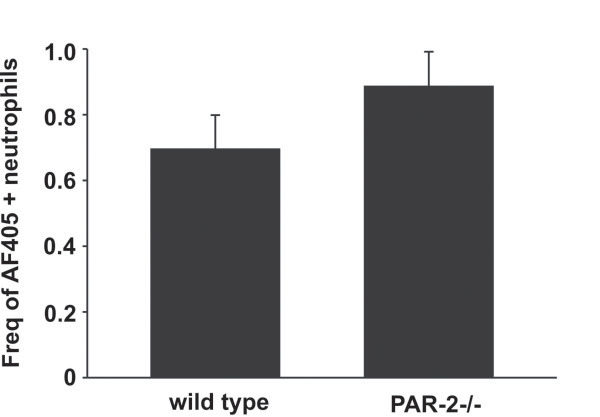
**PAR-2 expression does not alter allergen uptake in pulmonary neutrophils.** AF405-labeled GC frass was instilled in the airways of wild type and PAR-2-deficient mice and 20 h post exposure, lungs were isolated and stained for flow cytometry. The percentage of AF405-positive pulmonary neutrophils (gated on CD11c+, CD11b+, Gr1+ cells) in the lung are shown and the data are expressed as the mean ± SEM (n = 8 mice per group).

To determine if pulmonary neutrophils expressed PAR-2 and if these levels were regulated by allergen exposure, we performed a single intratracheal instillation of GC frass into naïve mice and harvested the lungs 20 h later. The lungs were digested and analyzed for PAR-2 expression by flow cytometry. In Figure 2A, a histogram represents the increase in PAR-2 levels in GC frass-treated pulmonary neutrophils compared to PBS treated cells. PAR-2 MFI on pulmonary neutrophils (Figure 2B) and PAR-2-positive neutrophils (Figure 2C) are also increased following GC frass treatment. Next, to determine if PAR-2 expression played a role in the uptake of GC frass, we exposed mice to a single intratracheal instillation of AF405-labeled GC frass and harvested the lungs 20 h later for flow cytometry. Gating on AF405-positive neutrophils, we found that wild type and PAR-2-deficient neutrophils had similar levels of AF405-positive neutrophils (Figure 3), suggesting that PAR-2 did not play a role in uptake of allergen in the lungs. In fact, only a small proportion of neutrophils had taken up AF405 at this time point. Together these data suggest that allergen exposure can upregulate the expression of PAR-2 on primary pulmonary neutrophils, but PAR-2 expression does not regulate allergen uptake in neutrophils.

### Activation of pulmonary neutrophils following GC frass exposure *in vivo*

**Figure 4:**
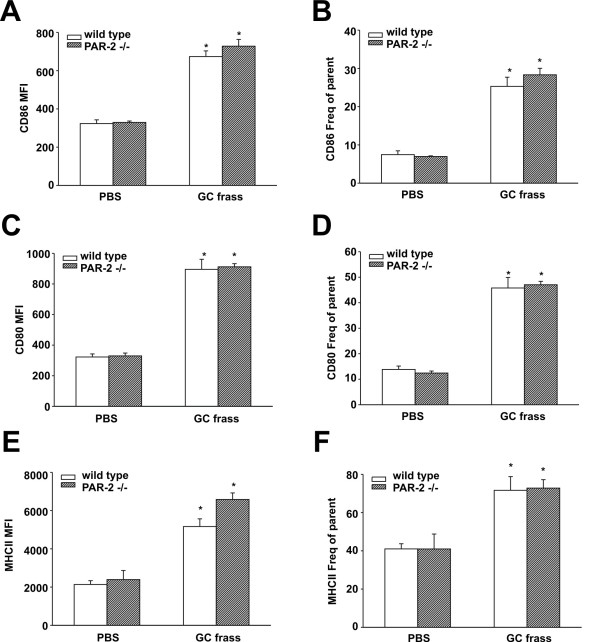
**Co-stimulatory molecule expression on pulmonary neutrophils in wild type and PAR-2-deficient mice following exposure to GC frass.** BALB/c and PAR-2-deficient mice were administered a single intratracheal instillation of PBS or GC frass and whole lungs were isolated 20 h later. Cells were dissociated from the lungs, and stained for flow cytometry analysis. Neutrophil (gated on CD11c+, CD11b+, Gr1+) populations were analysed for CD86 (**A** + **B**), CD80 (**C** + **D**), and MHC class II (**E** + **F**). MFI is shown in the left panel and the frequency of parent is shown on the right panel. Values are means ± SEM, n = 8 mice per group and statistical analysis was performed by ANOVA (*p < 0.001).

We asked whether levels of co-stimulatory molecules expressed on neutrophils were altered following GC frass treatment. Neutrophils express CD80, CD86, and MHC class II [12], all of which are important for activation of T cells. We measured the levels of CD80, CD86 and MHCII on pulmonary neutrophils following a single exposure to GC frass. We found that *in vivo* exposure to GC frass resulted in increased expression of these co-stimulatory molecules, both MFI and frequency of parent on wild type neutrophils (Figure 4). There was no difference in the increased levels of CD86, CD80, or MHCII between wild type and PAR-2-deficient mice, suggesting that allergen-associated proteases do not play a role in co-stimulatory molecule upregulation following allergen exposure.

### Ability of pulmonary neutrophils to respond to GC frass

**Figure 5:**
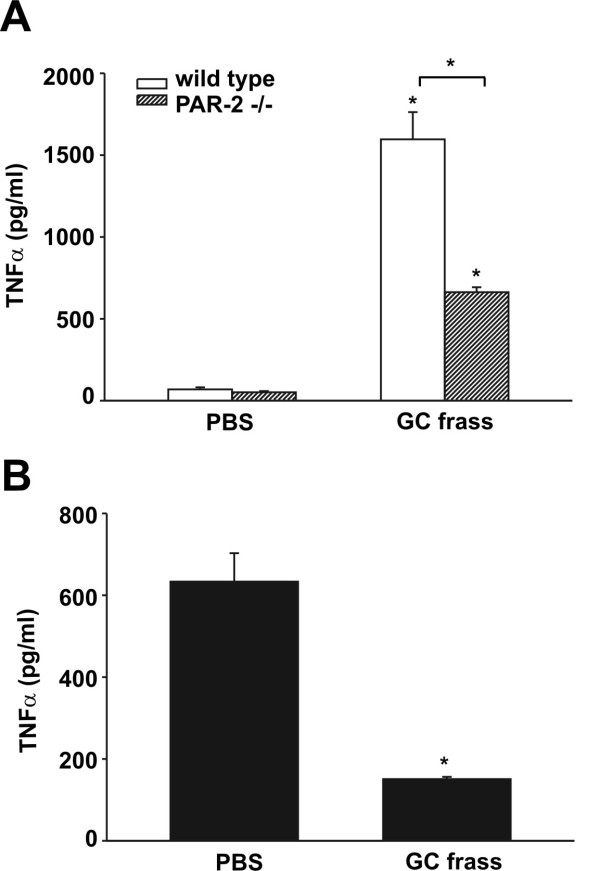
**TNFα release is diminished in pulmonary neutrophils from PAR-2-deficient mice compared to wild type mice. A**. Whole lungs from naïve BALB/c and PAR-2 mice were dissociated and neutrophils were isolated by Percoll gradient. Neutrophils (1 × 10^6^) were then treated *ex vivo* with PBS or GC frass and 18 h later, the supernatants were harvested, clarified, and analyzed for TNFα levels by ELISA. Data are expressed as means ± SEM (n = 3 separate experiments run in duplicate) and statistical significance was analyzed by ANOVA (*p < 0.001). **B**. BALB/c and PAR-2-deficient mice were administered a single intratracheal instillation of GC frass and 18 h later BAL fluid was harvested. Neutrophils (5 × 10^5^) were isolated by Percoll gradient and were cultured without additional treatment for 6 h. Supernatants were then harvested, clarified and analyzed for TNFα levels by ELISA. Data are expressed as means ± SEM (n = 5 mice per group) and statistical significance was analyzed by Student’s t-test (*p = 0.002).

We have previously shown that TNFα and IL-6 cytokine levels are significantly decreased in the BAL fluid following a single exposure of GC frass in PAR-2-deficient mice compared to wild type mice [10]. We wanted to determine if neutrophil-derived cytokine production from wild type and PAR-2-deficient mice was altered. To begin our investigation, we isolated lungs from naïve BALB/c and PAR-2-deficient mice and isolated the neutrophils on a Percoll gradient. We cultured equal numbers of neutrophils and treated these cells *ex vivo* with GC frass. We found that frass induced significantly more TNFα from neutrophils isolated from lungs of wild type mice compared to PAR-2-deficient mice (Figure 5A). To confirm cytokine production in pulmonary neutrophils in response to GC frass, we performed a single intratracheal instillation of GC frass into wild type and PAR-2-deficient mice and harvested the neutrophils from the BAL fluid by Percoll gradient. We then assayed these cells directly for cytokine production. We found a greater amount of TNFα production from BAL-derived neutrophils from wild type mice than from PAR-2-deficient mice (Figure 5B). Together these data show that TNFα production from neutrophils is mediated in part by the activation of PAR-2, and suggests a role for PAR-2 activation in initiating the innate immune response.

## Discussion

In this report, we investigated the role of neutrophils in the innate immune response to allergen, specifically related to the role of allergen-derived proteases and PAR-2. Our results suggest that allergen exposure upregulated PAR-2 on pulmonary neutrophils and that PAR-2 activation resulted in neutrophil-derived TNFα release. We have previously reported that a very early innate immune response occurred following allergen exposure. Within one hour, a significant release of TNFα in the BAL fluid is detected [13] and while this is maximal at 6 h post exposure, levels remain significantly higher than PBS controls up to 24 h later [7]. Neutrophil-derived TNFα production is important as we have shown that depletion of neutrophils prior to allergen exposure abolished GC frass-induced TNFα production when assessed 18 h later [7]. To our knowledge, this is the first study to address the role of cytokine production from PAR-2-deficient pulmonary neutrophils. Expression of PAR-2 was important for maximal expression of TNFα from pulmonary neutrophils. While we did not directly study this, it is likely that PAR-2-mediated TNFα production from neutrophils is mediated by nuclear factor (NF)-κB and extracellular regulated kinase (ERK). We have recently demonstrated that activation of PAR-2 regulated TNFα production by NF-κB and ERK, but not p38, in alveolar macrophages [10]. Other reports have also shown that PAR-2 activation leads to increased ERK and IκBα/NF-κB signal transduction pathway activation [14-16]. In our study, we report that the major consequence of PAR-2 activation on neutrophils is the release of TNFα in the airways.

Vergnolle et. al. showed that PAR-2 contributed to the early events of inflammation by playing a crucial role in leukocyte recruitment and extravasation [17]. In that study, selective activation of PAR-2 significantly increased leukocyte rolling and leukocyte adhesion to the endothelium. A subsequent study showed that leukocyte rolling was significantly lower in PAR-2-deficient mice compared to wild type mice in a model of acute inflammation [18]. In this report, we found that PAR-2-deficient mice were less responsive to GC frass in their ability to recruit neutrophils into the lungs and BAL fluid; however while this was statistically significant, the levels of neutrophils in the PAR-2-deficient mice compared to wild type were not completely repressed. While it is currently unclear from the previous studies [17,18] whether PAR-2 expression was crucial on the leukocyte or the endothelium, it is clear that PAR-2-deficient mice have a somewhat altered ability of neutrophil recruitment into the lungs and airways following allergen exposure. It is also important to note that in the PAR-2-deficient mice, we have previously reported a decrease in the neutrophil chemoattractant KC in the BAL fluid of mice following allergen exposure [10], which could also play an important role in neutrophil recruitment into the lung. These data are similar to those presented by Williams et al. [19] who found that KC levels in the BALF of PAR-2 mice were significantly lower following LPS than in wild type mice. Interestingly we found that MIP-2 release was unaltered in the PAR-2-deficient mice 18 h post allergen exposure. A recent report found an early and transient regulation of MIP-2, where MIP-2 release was reached a peak at 3 hr post inhalation and reached basal levels by 12 hr. In that study, the presence of PAR-2 regulated MIP-2 expression only at 3 hr post LPS exposure in the lung homogenate but not in the BALF [19]. It is currently unclear of the role MIP-2 would play in mediating neutrophil recruitment into the airways. Our current study cannot clearly identify if PAR-2 plays a role in extravasation of the neutrophil or the chemoattraction of neutrophils into the lung and thus further studies are required to answer this question.

Recently it was shown that neutrophils may act as professional antigen-presenting cells (APC) by inducing their expression of MHC class II and co-stimulatory molecules CD80 and CD86 and by processing and presenting antigen to trigger T-cell activation [12]. Based on that study, we investigated the levels of MHC class II, CD80 and CD86 on pulmonary neutrophils following a single exposure to GC frass and found that the lack of PAR-2 had no effect on the expression of these molecules. We have recently reported that on pulmonary mDCs, the expression of CD80 and CD86 was somewhat dependent on the presence of a functional PAR-2 [20]. In PAR-2-deficient mDCs, the expression of these co-stimulatory molecules were slightly, but significantly decreased. PAR-2 activation has been shown to enhance the maturation of bone marrow-derived DCs [21] suggesting the potential role for PAR-2 activation on the development of DCs as APC. It is still not completely clear what the overall relevance of neutrophils as APC has on the initiation of allergic airway inflammation, and further studies are needed in this area. However, in this report, we find that PAR-2 expression did not appear to be important for allergen uptake or phagocytosis of the allergen, nor did it appear to play a role in co-stimulatory molecule regulation on the neutrophil.

Many cells are likely involved in the initiation of the innate immune response, including the neutrophil, which we have consistently seen in high numbers following allergen exposure [7,10,13]. It is still unclear what the overall role of the neutrophil is in the initiation of allergic airway inflammation. In a guinea pig model of OVA-induced asthma, removal of neutrophils was found to decrease mucus production by preventing goblet cell degranulation [22]. Neutrophils also release interleukin (IL)-8, growth-related oncogene α (GRO-α), macrophage inflammatory protein 1-α (MIP-1α) and MIP-1β [23]. IL-8 and GRO-α act to recruit additional neutrophils, while MIP-1α and MIP-1β are chemoattactive for immature DCs and T cells. Thus, the neutrophil can play a direct role in altering the cellular milieu following stimulation.

We found that pulmonary neutrophils release a substantial amount of TNFα following allergen exposure. We showed this in two ways, first by isolating neutrophils from whole lung and treating them *ex vivo* with allergen, and second by directly isolating pulmonary neutrophils from BAL fluid following allergen exposure. The second method is more physiologically relevant in that we identified the amount of TNFα release from airway-derived neutrophils stimulated with GC frass *in vivo*. For this experiment, we counted the cells and cultured them in equal quantities, so it is likely that since there are less neutrophils in the airways of PAR-2 mice, the overall amount of neutrophil-derived TNFα in the airways will be even less. In a previous study, we found higher levels of TNFα in the BAL fluid of mice following allergen exposure so it is likely that other lung cells are also involved in TNFα release. Recently, TNFα was shown to enhance TGF-β1-driven epithelial-to-mesenchymal transition suggesting that TNFα could play a crucial role in the reprogramming of epithelial cell responses [24]. In patients with asthma, increased TNFα levels have been detected in the airways [25], and there is some evidence that increased airway TNFα may play a role in refractory asthma [26]. It is unclear what the major cellular source of this TNFα is, however there are a subset of asthma patients with refractory asthma that have significantly increased levels of neutrophils [27]. What is still unclear is the direct role of neutrophil-derived TNFα in modulating allergic airway inflammation.

The importance of PAR-2 in modulating allergic airway inflammation has recently been shown. PAR-2 mice exhibit decreased airway hyperresponsiveness, serum IgE and Th2 cytokine production following allergen sensitization and challenge compared to wild type controls [4,28]. Our collective data has shown an important role for GC frass-associated proteases and PAR-2 in modulating cytokine production from alveolar macrophages [10], airway epithelium [9], myeloid dendritic cells [20], and in the current study, neutrophils; all of which are important activators of the innate immune response. To our knowledge, this is the first report investigating the role of PAR-2 in mediating the activation of airway neutrophils following exposure to an allergen.

## Abbreviations

AF: Alexa Fluor; BAL: Bronchoalveolar lavage; CD: Cluster of differentiation; GC: German cockroach; MFI: Mean fluorescence intensity; MHC: Major histocompatibility complex; NF-κB: Nuclear factor κB; PAR: Protease activated receptor; TNF: Tumor necrosis factor.

## Competing interests

The authors declare that they have no competing interests.

## Authors’ contributions

RL participated in the design and implementation of the experiments and drafted the manuscript. IPL performed and analyzed the flow cytometry experiments. PZ performed all the cell work and isolated the neutrophils. JRL performed the animal studies and ELISAs. KP conceived of the study, participated in its design and coordination, and drafted the manuscript. All authors read and approved the final manuscript.
